# Predicting Students’ Academic Performance with Conditional Generative Adversarial Network and Deep SVM

**DOI:** 10.3390/s22134834

**Published:** 2022-06-26

**Authors:** Samina Sarwat, Naeem Ullah, Saima Sadiq, Robina Saleem, Muhammad Umer, Ala’ Abdulmajid Eshmawi, Abdullah Mohamed, Imran Ashraf

**Affiliations:** 1 Department of Humanities and Social Sciences, Khwaja Fareed University of Engineering and Information Technology, Rahim Yar Khan 64200, Pakistan; samina.sarwat@kfueit.edu.pk (S.S.); dr.naeemullah@kfueit.edu.pk (N.U.); rubinasaleem990@gmail.com (R.S.); 2 Department of Computer Science, Khwaja Fareed University of Engineering and Information Technology, Rahim Yar Khan 64200, Pakistan; s.kamrran@gmail.com; 3 Department of Computer Science Information Technology, The Islamia University of Bahawalpur, Bahawalpur 63100, Pakistan; 4 Department of Cybersecurity, College of Computer Science and Engineering, University of Jeddah, Jeddah 21959, Saudi Arabia; aaeshmawi@uj.edu.sa; 5 Research Centre, Future University in Egypt, New Cairo 11745, Egypt; mohamed.a@fue.edu.eg; 6 Department of Information and Communication Engineering, Yeungnam University, Gyeongsan 38541, Korea

**Keywords:** educational data, CGAN, SVM, predicting student performance, tutoring

## Abstract

The availability of educational data obtained by technology-assisted learning platforms can potentially be used to mine student behavior in order to address their problems and enhance the learning process. Educational data mining provides insights for professionals to make appropriate decisions. Learning platforms complement traditional learning environments and provide an opportunity to analyze students’ performance, thus mitigating the probability of student failures. Predicting students’ academic performance has become an important research area to take timely corrective actions, thereby increasing the efficacy of education systems. This study proposes an improved conditional generative adversarial network (CGAN) in combination with a deep-layer-based support vector machine (SVM) to predict students’ performance through school and home tutoring. Students’ educational datasets are predominantly small in size; to handle this problem, synthetic data samples are generated by an improved CGAN. To prove its effectiveness, results are compared with and without applying CGAN. Results indicate that school and home tutoring combined have a positive impact on students’ performance when the model is trained after applying CGAN. For an extensive evaluation of deep SVM, multiple kernel-based approaches are investigated, including radial, linear, sigmoid, and polynomial functions, and their performance is analyzed. The proposed improved CGAN coupled with deep SVM outperforms in terms of sensitivity, specificity, and area under the curve when compared with solutions from the existing literature.

## 1. Introduction

The rapid development of technology and wide deployment of technology-assisted educational platforms opened new paradigms for the education system, especially in the context of the COVID-19 era, which stopped the operations of traditional educational systems. Such platforms have the potential to monitor students’ activities and collect data that can be used to investigate students’ problems and take corresponding actions in time. Preventive and corrective actions can thus reduce the probability of students’ failure and enhance the performance of academic institutions. Educational data mining (EDM) specifically deals with such problems and helps both instructors and students. EDM is a burgeoning field of study that examines educational data for a variety of academic tasks. The most common use of EDM is to predict students’ academic performance. The analysis and interpretation of students’ performance have been regarded as the most important topic in academics, and it requires appropriate analysis, assessment, and evaluation techniques. In today’s knowledge-based economy, students are an important asset for a country’s socio-economic growth. Therefore, keeping students’ performance on track is critical [1]. A variety of information and communication technology (ICT)-based learning methods have been used in higher education institutions (HEIs). In these methods, various learning environments are leveraged to make the learning process easier and disseminate knowledge to the students in the most favorable way [2]. Furthermore, these environments keep a record of users and users’ interactions within the environment for auditing and recovery purposes.

Learning management systems and other similar learning resources are used for HEIS students that operate using the internet. Data from such computer-based learning systems can be logged and used later. For example, LMS [3], student logs (Moodle) [4], and video interactions [5] are frequently used in an educational context, and their data are used to assess students’ academic performance. The use of these systems in educational institutes produces a large amount of data that can be utilized for further analysis to investigate different factors that can be influential in monitoring students’ performance and carrying out corrective actions where needed. Data analysis to monitor students’ performance can play an important role in boosting their academic performance and improving teaching quality [6]. However, analyzing such a large amount of data is not trivial and can be laborious if done manually. Machine learning algorithms for predicting student academic performance is an emerging field in educational data mining that can potentially solve this problem [7].

Many studies have employed machine learning models to predict student performance using EDM [8,9]. However, these studies mainly focus on demographic data, and prediction has been performed based on online activities. Nevertheless, only a few researchers have worked on analyzing the video interactions of learners in a video-assisted course [10,11]. Similarly, the impact of school and home tutoring is also an under-investigated area regarding LMS data. This study predicts the educational impact of success/failure and performs binary classification (pass/fail). In this regard, this study proposes an approach and makes the following contributions:

- The study proposes a novel improved conditional generative adversarial network (CGAN). The model is augmented by a support vector machine (SVM), which improves its performance further.
- In comparison, several machine learning models are also utilized, such as logistic regression (LR), extra tree classifier (ETC), gradient boosting machine (GBM), and stochastic gradient descent (SGD). In addition, convolutional neural network (CNN) and long short-term memory models are also implemented for performance comparison.
- The study uses data from different online sources such as Moodle, SIS, and eDify (a video-assisted course) that can help to find and understand video learning analytics using educational data mining. The prediction performance is evaluated regarding different tutoring methods, including ‘school tutoring’, ‘home tutoring’, and ‘combined tutoring’.

The paper is further arranged as follows. Section 2 provides related work. Section 3 discusses the datasets and methods used in the proposed framework. Section 4 presents results and discussion. In the end, Section 5 provides a conclusion and future directions.

## 2. Related Work

Educational data mining is booming due to the rapid growth of the internet, educational resources, and the use of online learning tools to provide education [12]. Consistent efforts are being done by researchers to assess students’ performance by improving technical educational supportive tools. This section discusses the elements that might affect student academic performance, as well as technologies that can assist in making predictions about students’ academic performance. For example, the probability of failure assessed at an early stage was studied by [13] using an SVM in a programming course. Online platforms have facilitated distance learning, and the use of LMS has also provided an opportunity to analyze the online activities of students. Moodle LMS has been analyzed by researchers to observe student behavioral patterns [14,15].

Nabil et al. [16] worked on predicting student academic performance based on course grades using deep neural networks. The study predicted students’ performance for upcoming courses by using the grades in previous courses. Performance was evaluated using machine learning models, such as LR, random forest (RF), decision tree (DT), k-nearest neighbors (KNN), support vector classifier (SVC), and GBM. To handle imbalanced class problems, the upsampling technique was used. The deep neural network achieved an accuracy value of 89%. Similarly, ref. [17] proposed a machine-learning-based intelligent decision support system for students’ performance in online learning. The authors used decision trees in RF, gradient boosting trees, naïve Bayes, and KNN. The performance of these algorithms was further improved by using ensemble techniques such as voting, stacking, boosting, and bagging. The F1 score of the individual models was good, but when stacking was performed by combining all the classifiers, an F1 score of 0.8195 was obtained.

The authors investigated the performance of e-commerce students in [18] with the objective of determining students’ performance at semester end using video analytics and data mining tools. In addition, the e-commerce technology modules at HEIs were also evaluated. An accuracy of 88.3% was achieved with an optimized RF. Oku et al. [19] used two machine learning algorithms to determine student engagement in performing multiple tasks during an online session. The LR and RF models were adopted and achieved accuracy scores of 66% and 63%, respectively.

An ensemble meta-based tree (EMT) model was proposed in [20] for the prediction of students’ academic performance. The authors used J48, decision table, multilayer perceptron (MLP), and NB algorithms, with bagging and boosting methods. A dataset of students’ LMS activities, such as students’ interactions with the online learning system, was used for this purpose. An accuracy of 80.33% was achieved using the MLP. Similarly, the authors worked on the factors that affect the student’s performance in [21]. The study used three machine learning algorithms to find that the economic background of the student is the main factor that affects their performance. Wrapper-based and filter-based feature extraction techniques were used to achieve 88% accuracy. Aggarwal et al. [22] considered academic and non-academic parameters for student performance analysis. The authors considered LR, RF, SVC, DT, bagging, boosting, voting, AdaBoost, and MLP for experiments. RF with SMOTE showed better results, with a 93.8% F1 score.

The authors compared the performance of several machine learning classifiers for prediction of students’ academic performance in [23]. LR, ANOVA, SV regression, DT regression, log-linear regression, RF regression, and partial least square regression were used in the study. The results indicate a better performance of the log-linear regression. Mubarak et al. [24] used LSTM on features obtained from video clickstream data. The study predicted the weekly performance of students, thus allowing timely corrective measures. The proposed LSTM outperforms baseline SVM, ANN, and LR, with an accuracy of 93%. To determine student dropout in self-paced MOOC courses, Dass et al. [25] proposed an RF-based approach. With an accuracy of 87.5%, the proposed system could predict the student dropout rate in the MOOC course. Ram et al. [26] proposed a machine learning system for the prediction of student academic performance using SVM, AdaBoost, LR, and RF classifiers. The study reported a 92% accuracy value using SVM and RF, and a 91% accuracy score using LR and AdaBoost.

Academic performance prediction is significant in determining indicators that can improve students’ performance and help policymakers to reduce students’ dropout ratio. It can also support students in improving academic behaviors and learning strategies. Different past studies have focused on different activities, such as determining the dropout ratio [27], CGPS [28], student interaction [29], demographic data [30], expenses and depression [31], and failure risk [32]. Students at failure risk were determined by using various machine learning models such as DT, LR, and NB [8]. The authors observed students’ activities with respect to their grades using MOOC data and claimed that aggregated frequency of activities has a significant impact on students’ grades. In another work [33], the authors applied DT, investigated factors that had a negative impact on their performance, and provided appropriate suggestions. Predominantly, such works consider socio-economic and demographic factors for predicting students’ academic performance, and attributes related to students’ interactions are under-investigated. Similarly, school and home tutoring factors are not very well studied. This study considers these attributes and devises a novel approach to predict students’ performance.

Some studies focus on parameter optimization for different machine learning algorithms. For example, ref. [34] combines active disturbance rejection control with proportional-derivative Takagi–Sugeno fuzzy control, which is tuned by virtual reference feedback tuning. The hybrid model can automatically tune the model for optimal performance. In addition, the proposed hybrid model requires less time for tuning. Similarly, ref. [35] presents an indirect iterative learning control mechanism to increase the performance of the P-type controller. Learning gain is improved in real time using I/O measurements.

## 3. Material and Methods

This section discusses the proposed approach, the dataset used for experiments, and a brief description of machine learning models used for performance comparison.

### 3.1. Dataset

The dataset to predict students’ performance is taken from [36] and contains the records for 786 students, of which 649 records belong to a Portuguese class, while the remaining 395 belong to a math class. Of the 33 total attributes, 9 attributes are related to home and school tutoring. Of the total attributes, 24 attributes are collected via questionnaires, while the rest are from reports from the school. A 20-point grading system is used, where ‘0’ represents the lowest grade and ‘20’ represents the highest grade. Students are evaluated three times during a semester, and the last grade (G3) is the final grade awarded to the students. A detailed description of the dataset is presented in Table 1, where the record of the last four rows has been taken from the school report.

This study uses the dataset from three perspectives. At first, only school tutoring is considered, followed by home tutoring, and both school and home tutoring are combined as the third choice.

### 3.2. Generation of Training Data by GAN

GAN is an emerging network model that has been largely used for unsupervised and semi-supervised learning. One network, known as a generator G, generates fake data samples similar to real data, while the other, known as discriminator D, is fed with both real and fake data samples to define real and fake. Both networks work simultaneously and strive to achieve Nash equilibrium. The generator network has no access to real data, and it can only interact through a discriminator that has both real and fake data samples. The discriminator creates an error signal based on the ground truth in determining whether the data generated by the generator is real or fake. The error signal is used to improve the performance of the generator in generating more fake data of good quality.

A multilayered network comprising fully connected or convolutional layers is generally used as a generator or discriminator. The generator should be distinguishable from the discriminator, but it is not necessary for it to be exactly invertible. Recent progress of GAN has been presented in several studies [37]. There are different types of GAN approaches, such as fully connected GAN, convolutional GAN, conditional GAN, inference model GAN, and adversarial autoencoder GAN. This study utilizes conditional GAN (CGAN), proposed by [38], which uses a conditional class for both generator and discriminator. Conditional GAN uses conditional variables to present multidimensional data generation in a better way and can handle the random noise generated in the original GAN. Various forms of conditional GAN have also been proposed, such as auxiliary GAN [39] and InfoGAN [40]. Existing CGAN has its own merits and demerits. This study proposes an improved CGAN that integrates the features of previous CGANs. Mathematically, it can be written as follows:

(1) $$L_{source}=E\left[logP\left(S=real|X_{real}\right)\right]+E\left[logP\left(S=fake|X_{fake}\right)\right]$$

(2) $$L_{class}=E\left[logP\left(C=a|X_{real}\right)\right]+E\left[logP\left(C=a|X_{fake}\right)\right]$$

(3) $$I\left(a,G\left(n,a\right)\right)=E_{x∼G(n,a)}\left[E_{a∼P(a|x)}\left[logQ\left(a|x\right)\right]\right]+H\left(a\right)$$

The intention of this formulation is to optimize $L_{source}$ + $L_{class}$ - λI(a,G(n,a)) for the discriminator and optimize $L_{class}$ - $L_{source}$- λI(a,G(n,a)) for the generator. Note that λ is the hyperparameter, and the information between G(n,a) and *a* is represented by I(a,G(n,a)).

**Figure 1:**
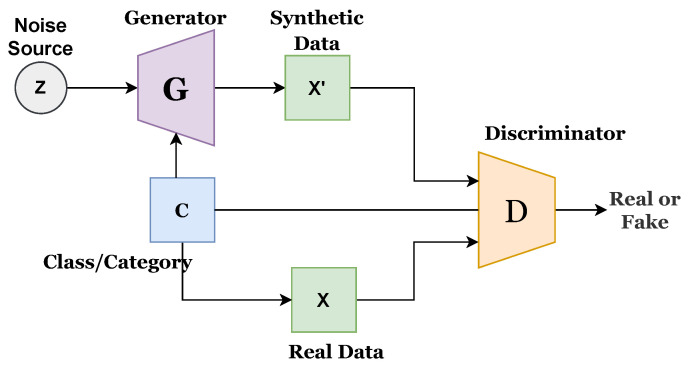
Architectural diagram of existing CGAN.

**Figure 2:**
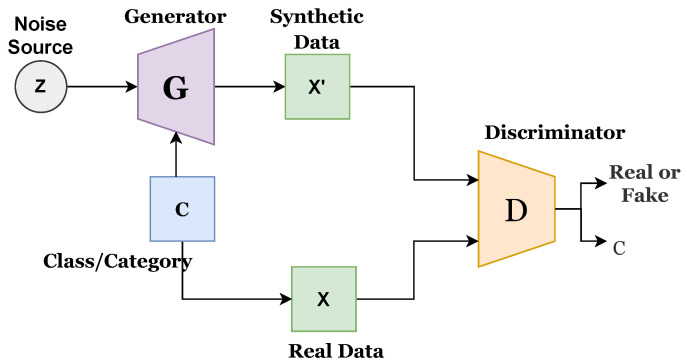
Architecture of existing InfoGAN model.

Figure 1 presents the architecture of the existing CGAN, while Figure 2 presents the existing infoGAN. The ‘z’ refers to the noise source, ‘c’ is the category or class, ‘G’ indicates the generator, ‘X’ is the real data, X’ presents synthetic or generated data, and ‘A’ indicates the additional network. G generates the synthetic data, and D predicts the probability score to determine the source of coming data, whether it comes from G or a real dataset. G and D both are conditional networks. Generated data could be biased, and this can be avoided by increasing the diversity of the data.

**Figure 3:**
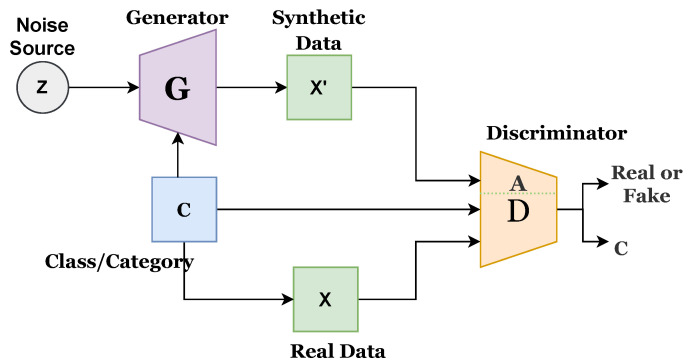
Architecture of proposed improved CGAN model.

Figure 3 presents the proposed improved CGAN. The proposed CGAN has made three modifications to the existing CGAN:

- Introduced conditional or class variable to D;
- Added an extended network with D; and
- Assigned a label to each sample of data.

### 3.3. Prediction of Students’ Performance

**Figure 4:**
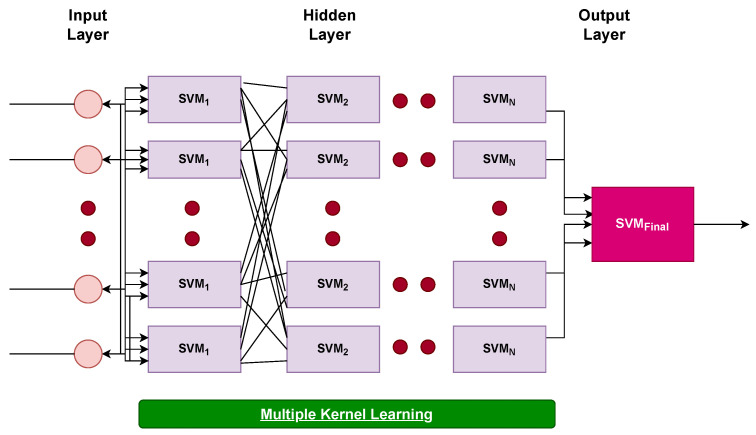
Architecture of proposed deep SVM.

The model designed for predicting students’ performance is based on deep SVM layers. The framework consists of several hidden layers of SVM, like a deep neural network model. However, the deep architecture of SVM is more flexible due to its kernel function estimation and can handle large-sized input vectors. It can perform better on small-sized datasets. The proposed SVM can avoid overfitting due to its powerful regularization ability. The architectural flow of deep SVM is presented in Figure 4. The number of hidden layers can vary according to training data. In this study, the grid search approach has been used for the selection of the number of hidden layers, which also reduces the computational complexity. An increase in the number of layers can also increase the computational complexity and decrease the model’s performance.

Generally, in the SVM model, kernels such as radial basis, linear, sigmoid, and polynomial have been utilized by existing studies. Because of the below-optimal performance of these kernels for many tasks, multiple customized kernels have also been used by several studies [41,42,43]. This work combines kernel functions to form a customized kernel using Mercer’s theorem [44]. The model performs better by taking advantage of kernel functions that are on a radial basis; linear, sigmoid, and polynomial kernels are shown in Equations (1)–(4), respectively.

(4) $$K_{1}\left(x_{1},x_{2}\right)=\left(x_{1},x_{2}\right)$$

(5) $$K_{2}\left(x_{1},x_{2}\right)=exp(||x_{1}-x_{2}{\left|\right|}^{2}/2\sigma )$$

(6) $$K_{3}\left(x_{1},x_{2}\right)={\left(x_{1},x_{2}+c\right)}^{p}$$

(7) $$K_{4}\left(x_{1},x_{2}\right)=tanh\left(x_{1},x_{2}+c\right)$$

where $k(x_{1},x_{2})$ is the kernel function, *p* is a positive number, and *c* is a real number.

For learning multiple kernels, a heuristic approach has been applied. By considering the mean square error, the M-heuristic is presented in Equation (5).

(8) $$\mu_{i}=\sum_{i=i}^{4}\left(M_{i}-M_{j}\right)/\sum_{j=i}^{4}\sum_{i=i}^{4}\left(M_{i}-M_{j}\right)$$

It is worth mentioning that in each layer of SVM, the design of multiple kernels may vary. The proposed approach is superior to those in the previous literature for two reasons. First, improved CGAN is comparable to the complexity of infoGAN and ACGAN, as it is based on these approaches. Second, SVM is less complex compared to deep learning models such as convolutional neural networks. A step-by-step instruction guide is presented in Algorithm 1 for implementation of the proposed approach:
**Algorithm 1** Deep SVM1:svcLinear = SVC(kernel=’linear’)2:svcPoly = SVC(kernel=’poly’, degree=8)3:svcGaussian = SVC(kernel=’rbf’)4:svcSigmoid = SVC(kernel=’sigmoid’)5:model = Sequential()6:model.add(svcLinear)7:model.add(svcPoly)8:model.add(svcGaussian)9:model.add(svcSigmoid)10:model.fit_generator(X_train, Y_train)

### 3.4. Supervised Machine Learning Algorithms

In this section, the machine learning algorithms used in this study are briefly discussed.

#### Random Forest

RF is a supervised learning algorithm that consists of many DTs working individually to predict the results of a class where the final prediction is based on the class that gets the majority of votes. The error rate for RF is relatively low compared to other models. The reason for the low error rate is that it has a low correlation between trees [45]. This study uses RF with different optimized parameters. Based on the problem, multiple algorithms are used to decide the split in the decision tree. Similarly, the maximum number of DTs is also set for optimal training.

### 3.5. Logistic Regression

LR is a statistical method in which one or more variables are used to compute the final result. LR is widely used to compute the probability of the class numbers; therefore, LR is the best learning model when the target class is categorical [46]. It processes the relationship among one or more variables and categorical independent variables by estimating the probabilities using logistic functions. LR uses the sigmoid function to transform the output into a probability value. The aim is to achieve the optimal probability with a low value of the cost function.

### 3.6. Extra Tree Classifier

ETC is an ensemble learning model. The working principle of ETC is quite similar to RF, and the only difference is in the construction of the trees in the forest. In ETC, every tree is built using the original training samples. Random samples of the k best features and Gini index are used to select the best features to split the data in the tree. This approach results in the construction of the de-correlated trees in ETC [47].

#### 3.6.1. Gradient Boosting Machine

GBM is based on boosting and is a powerful ensemble model extensively used to handle classification problems. In GBM, many weak classifiers work together to form a strong learning model. It usually works on the principle of the DT [48]. GBM creates every tree independently, so it is an expensive and time-consuming choice. Due to the high probability of approximation of correct learning, it works well on the unprocessed data. To deal with the data’s missing values, GBM is a good choice.

#### 3.6.2. Stochastic Gradient Descent

The working of SGD is based on the working principle of logistic regression convex loss function and SVM. It is a good choice for multi-class classification problems because it combines multiple binary classifiers and the one versus all method. SGD works well on large datasets because it takes this idea to the extreme. SGD uses a single sample in an iteration. It is easy to understand and easy to implement the regression model. The hyperparameters of SGD need to be selected and optimized appropriately to obtain good results [49].

### 3.7. Deep Learning Models

In addition to machine learning models, two deep learning models are also deployed for performance comparison: CNN and LSTM. A brief description of each is provided here.

#### 3.7.1. Convolutional Neural Network

CNN is a deep neural network that is widely used for image classification tasks. It learns the complex features associated with the target class during training efficiently [50]. CNN is composed of several types of layers, such as convolutional, pooling, activation, and flatten; dropout layers are also used. Features are learned from the input data at the convolutional layer, while the pooling layer reduces the size of extracted features and lowers the computational complexity. Max-pooling is used in this study for experiments. The dropout layer aims at reducing the probability of overfitting, while the flatten layer transforms the data into an array. The rectified linear unit is applied as an activation function in this study, and the dropout rate is 0.2.

#### 3.7.2. Long Short Term Memory Network

A recurrent neural network (RNN) is a feed-forward deep neural network model that faces vanishing gradient problems and loss of information in dealing with long sequences of information. LSTM is an extended form of RNN. LSTM saves information and deals with long sequences effectively by using memory cells and three gates. It uses structured gates to add or forget information to control memory cells. Forget gate is used to decide which information is to be removed [51]. The sigmoid function is used for this purpose: if the output is 1, information is remembered, while it forgets the information if the output is 0. This is performed based on the current state and previous state.

## 4. Results and Discussions

This section presents the results and discussion of the results for student performance prediction.

### 4.1. Experimental Setup

For implementation of the machine learning models, the SciKit-learn library and Natural Language Process Tool Kit (NLTK) is used. Machine learning algorithms are deployed in Python using the SciKit module. Jupyter notebook is used for performing experiments.

### 4.2. Performance Evaluation Metrics

In this section, the results of the proposed work are presented from different perspectives. For example, the performance of the proposed approach is evaluated on two datasets regarding students’ academic performance prediction. Results are compared using improved CGAN and not using improved CGAN with deep SVM. Comparison has also been performed on multiple kernels and individual kernels. The performance of the proposed model is evaluated based on sensitivity, specificity, and AUC, using the following equations:

(9) $$\mathrm{Sensitivity}=TN/N_{n}$$

(10) $$\mathrm{Specificity}=TP/N_{p}$$

(11) AUC=Specificity−(1−Specificity)

### 4.3. Performance of the Proposed Approach without Improved CGAN

Student performance is predicted regarding three types of tutoring methods, which are school tutoring, home tutoring, and combined tutoring methods. The number of hidden layers has been selected using a grid search approach. Experiments are performed using one to six hidden layers, and the best results are obtained using three hidden layers. Therefore, only the results using three hidden layers are discussed.

Table 2 presents the performance of deep SVM without use of the proposed improved CGAN approach. Results indicate that the values for sensitivity, specificity, and AUC vary with respect to the tutoring method. The lowest performance to predict students’ performance is obtained when school tutoring attributes are used with deep SVM, where 91.8% each for sensitivity and specificity and 90.9% ACU is obtained. The performance using the attributes related to home tutoring is comparatively better, and sensitivity is increased to 92.2%. However, the best prediction results can be obtained when the attributes of the home and school tutoring are used in combination. The highest performance in terms of sensitivity, specificity, and AUC with 95.2%, 94.7%, and 92.9%, respectively, are obtained using combined tutoring.

### 4.4. Results of Proposed Approach with Improved CGAN

Table 3 presents the performance of deep SVM after applying improved CGAN. Results indicate that the performance has been increased substantially, except for the home tutoring case, where the sensitivity is reduced by a margin of 0.03%. It can be observed that the use of improved CGAN for data generation improves the sensitivity, specificity, and AUC for school and combined tutoring cases. School tutoring has shown better results compared to home tutoring. The best results have been achieved by the combined tutoring method with 98.2% sensitivity, 97.1% specificity, and 96.2% AUC. On the other hand, the sensitivity of school tutoring has increased from 91.8% to 95.1%, similar to the specificity and AUC scores.

Results reveal that the proposed model performs best when attributes from school and home tutoring are combined. Since both school and home tutoring help emphasize students’ better learning, combining the attributes from these methods provides appropriate features to predict students’ performance. Similarly, comparing school tutoring with home tutoring, the performance is almost similar when deep SVM is used without CGAN. However, the prediction is better for school tutoring when improved CGAN is utilized. School tutoring is better than home tutoring because school teachers are mostly experts and experienced.

Experimental results of deep learning models CNN and LSTM using the improved CGAN are given in Table 4. Results show similar trends for sensitivity, specificity, and AUC to that of deep SVM with the difference in performance. The performance of CNN and LSTM is higher for school tutoring as compared to home tutoring. However, the highest performance is obtained for combined tutoring. CNN shows better results than LSTM with the highest sensitivity of 97.3% for combined tutoring, 92.5% for school tutoring, and 89.9% for home tutoring. Similarly, the values of CNN for specificity and AUC are also higher. Despite the good performance of CNN, its performance is still inferior to the proposed deep SVM which obtains a 98.2% sensitivity for the combined tutoring in comparison to the 97.3% sensitivity of the CNN.

### 4.5. Influence of Number of Hidden Layers

**Figure 5:**
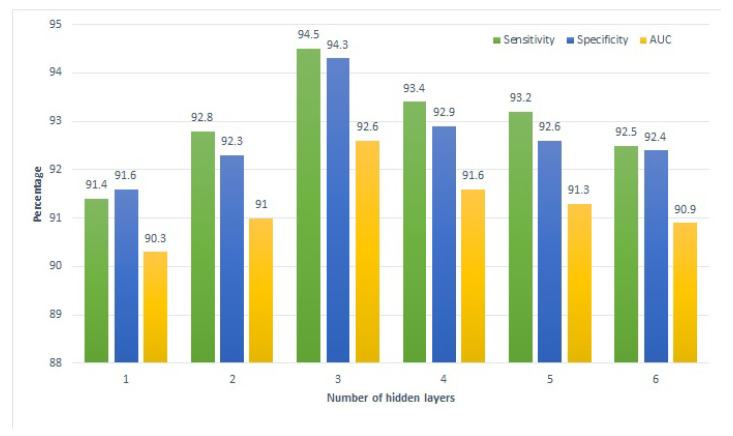
Performance of the proposed approach on school tutoring.

The influence of the number of hidden layers used in the deep SVM is also investigated to determine the optimal performance of the model. For this purpose, the hidden layers vary between one and six. The same process is carried out for school, home, and combined tutoring. Figure 5 shows the performance of school tutoring for each hidden layer regarding sensitivity, specificity, and AUC. It shows that the performance is gradually increased when we increase the number of hidden layers from one to three. However, after that, the performance is decreased with the increment of each hidden layer. Although the decrease is gradual, the performance of SVM with six hidden layers is still better compared to one layer.

**Figure 6:**
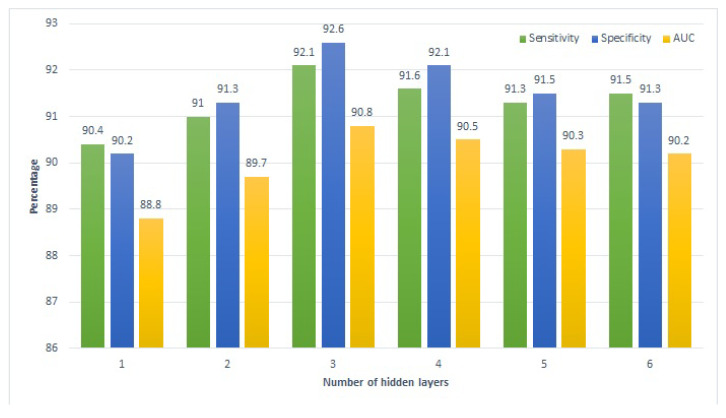
Performance of the proposed approach on home tutoring.

**Figure 7:**
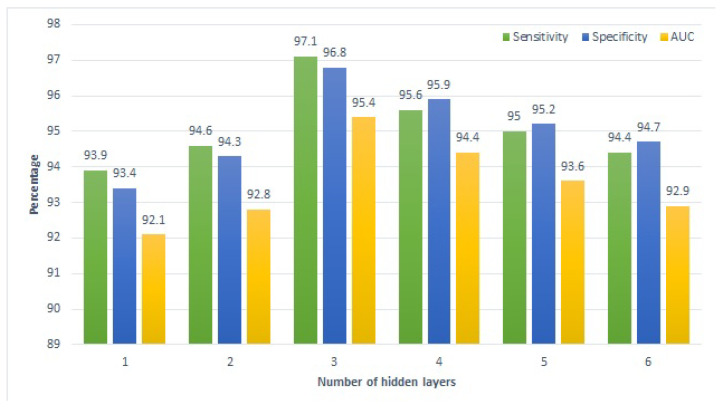
Performance of the proposed approach on combined tutoring.

Similarly, Figure 6 and Figure 7 present the performance of the proposed approach with a varying number of hidden layers for the home tutoring and combined tutoring cases, respectively. A pattern similar to that of school tutoring is observed for the performance of deep SVM here. Performance metrics show a gradual rise with the increase in the number of hidden layers from one to three, while increasing the layers further leads to a negative impact on the model’s performance. In a nutshell, all approaches show the best performance using three hidden layers.

### 4.6. Selection of Appropriate Kernel Type

Existing studies show that the choice of the kernel for SVM has a substantial impact on its performance [41,42,43]. In addition, modified and custom kernels have been utilized for better results. For increasing the effectiveness of the use of multiple kernel-based approaches, experiments have been performed using typical kernel functions such as radial, linear, polynomial, and sigmoid. Additionally, multiple kernels have also been used to investigate the impact on the model’s performance.

**Figure 8:**
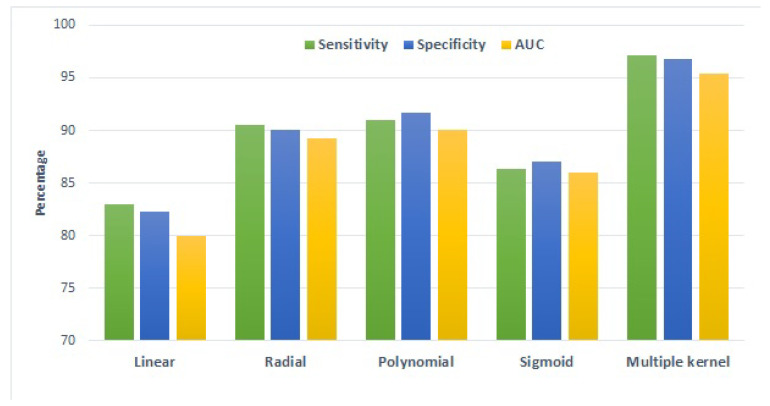
Performance of the proposed approach using multiple kernels and typical kernel functions.

Results are presented in Figure 8, which indicates that the best results are obtained using the polynomial kernel when a single kernel is used. However, when using multiple kernels, better performance can be achieved. It can be observed that the use of multiple kernels significantly improved the results in terms of the used evaluation metrics. Hence, multiple kernels improved the performance of the proposed approach by taking advantage of each kernel.

### 4.7. Performance Comparison with Machine Learning Models

A comparison of the proposed deep SVM is carried out with other selected machine learning models. These models are selected with regards to the results reported in the existing literature [52,53,54]. From this perspective, RF, LR, ETC, GBM, and SGD are implemented for performance comparison. These models are used for performance prediction using the three cases of school, home, and combined tutoring, similar to experiments using the proposed deep SVM model.

Table 5 shows the results for the school tutoring case. Results suggest that the best performance is obtained using LR, with 92.2% and 92.8% for sensitivity and AUC, respectively. ETC obtains the highest specificity of 93.4% for school tutoring. RF and ETC have similar sensitivity of 91.3%, while their AUC values are 91.8% and 91.5%, respectively. Results confirm that the proposed SVM shows much better results for school tutoring cases, with 95.1% for both sensitivity and specificity and 93.2% for AUC.

Results for the home tutoring scenario are provided in Table 6, which indicates that the performance of machine learning models has degraded. The highest sensitivity of 89.7% is obtained by ETC, followed by SGD and RF. Regarding specificity and AUC, the performance of ETC and SGD is superior to other models. Still, deep SVM shows far better results for home tutoring, where its sensitivity, specificity, and AUC are 91.9%, 91.2%, and 91.4%, respectively.

Table 7 presents the results of machine learning models regarding the combined tutoring case. Results are significantly improved when combined tutoring is applied. SGD shows superior performance regarding sensitivity, with 95.8%, followed by ETC and RF. The highest specificity of 95.4% and AUC of 94.5% are obtained by RF. Despite such good results from machine learning models, the performance of the proposed deep SVM with improved CGAN is superior with 98.2%, 97.1%, and 96.2% for sensitivity, specificity, and AUC, respectively.

### 4.8. Validation of Proposed Approach

An additional dataset is used in this study to validate the significance of the proposed approach. The dataset is obtained from Hasan et al. [55], who investigate use of the eDify mobile application to enhance the teaching and learning of students and their performance evaluation. The dataset consisted of three categories: students’ academic information, students’ activities, and students’ video interactions. The ‘students’ academic information’ data are extracted from the student information system, ‘students’ activity data’ from MOODLE, and ‘student’s video interactions’ from the eDify mobile application. The final dataset has 21 features and 326 instances. The features are ‘applicant name’, ‘CGPA’, ‘attempt count’, ‘remote student’, ‘prohibition’, ‘high risk’, ‘term exceeded’, ‘at risk’, ‘at risk SSC’, ‘other modules’, ‘plagiarism history’, ‘CW1’, ‘CW2’, ‘ESE’, ‘online c’, ‘online O’, ‘played’, ’paused’, ‘likes’, ‘segment’, and ‘result’. For experiments, the same process is followed that was used with the first dataset. Results indicate that the proposed approach can obtain good results with this dataset as well. The sensitivity, specificity, and AUC of the deep SVM using three hidden layers along with improved CGAN are 92.67%, 91.25%, and 91.25%, respectively.

### 4.9. Comparison with Existing Studies

**Figure 9:**
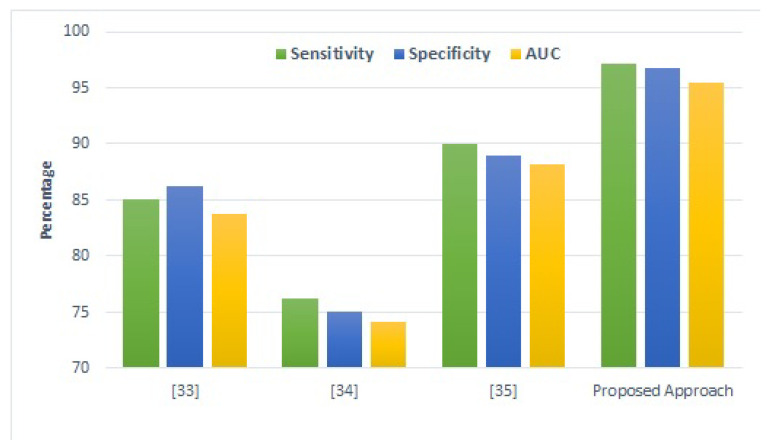
Performance comparison with existing state-of-the-art approaches. (Note that reference numbers in the figure are wrong).

For further corroboration and robustness of the proposed approach, results are compared with the best performing approaches from the existing literature on student performance prediction. Figure 9 presents a comparison of the proposed model with those from the previous literature. It shows that the proposed model is the best performing model. The proposed model shows superior results compared to existing approaches [56,57]. Besides these studies, ref. [58] used a deep learning model that is most suitable for large-sized datasets. As the datasets used in this study are comparatively smaller, their performance is poor.

The proposed approach does show superior results, especially for small-sized datasets. The main reason behind the best performance of the proposed approach is that improved CGAN deals with the problem of the low volume of data by generating synthetic data samples and improved the performance of deep SVM. Furthermore, the use of the multiple kernel approach also took advantage of different kernels and assisted deep SVM in achieving the best performance for predicting students’ performance.

The proposed model uses conditional GAN for training, which is designed to overcome the limitations of its discriminative counterparts. However, CGAN also has limitations. For example, it generates good data if learned space is used for mapping input. If a generator finds a data sample that can easily fool the discriminator, then it produces similar data and causes problems in training. In such cases, the model produces poor results for the unseen data.

## 5. Conclusions

With the availability of a large amount of data regarding students’ interactions with learning management systems, the use of educational data mining has become a potential tool to analyze and predict students’ academic performance and help them improve their performance by taking timely appropriate actions. Existing studies predominantly make use of demographic attributes from such systems, and other attributes are less investigated. This study leverages attributes related to school, home, and combined tutoring to predict students’ academic performance. Following a deep learning architecture, this study proposes a deep SVM that is augmented by an improved CGAN model. The performance of deep SVM is further enhanced by utilizing multiple kernels. Experiments are performed from several perspectives, such as the use of single vs. multiple kernels, the number of layers for deep SVM, deep SVM with and without improved CGAN, machine learning models, and CNN and LSTM deep learning models. Results indicate that multiple kernel learning is better than single kernels and deep SVM produces the best results using three layers. Performance analysis with the machine and deep learning models suggests that the deep SVM outperforms all models, with 98.2% accuracy, 97.1% specificity, and 96.2% AUC using the combined tutoring method. Comparing its performance with existing studies further corroborates its robustness and superior performance. The findings of this research can help tutors, students, teachers, and parents in choosing the best support for student learning during their educational period.

## Figures and Tables

**Table 1:** Attributes of students from dataset.

Attribute	Description	Domain
sex	student’s gender	binary: female or male
age	student’s age	numeric: from 15 to 22
school	student’s school	binary: Gabriel Pereira or Mousinho da Silveira
address	student’s home address	binary: urban or rural
Pstatus	parent’s cohabitation status	binary: living together or apart
Medu	mother’s education	numeric: from 0 to 4
Mjob	mother’s job	nominal
Fedu	father’s education	numeric: from 0 to 4
Fjob	father’s job	nominal
guardian	student’s guardian	nominal: mother, father, or other
famsize	family size	binary: ≤3 or >3
famrel	quality of family relationships	numeric: from 1—very bad to 5—excellent
reason	reason to choose this school	nominal: close to home, school reputation, course preference, or other
traveltime	home to school travel time	numeric: 1 ≤ 15 min, 2–15 to 30 min, 3–30 min. to 1 h or 4 ≥ 1 h
studytime	weekly study time	numeric: 1 ≤ 2 h, 2–2 to 5 h, 3–5 to 10 h or 4 ≥ 10 h
failures	number of past class failures	numeric: n if 1 ≤ n < 3, else 4
schoolsup	extra educational school support	binary: yes or no
famsup	family educational support	binary: yes or no
activities	extra-curricular activities	binary: yes or no
paidclass	extra paid classes	binary: yes or no
internet	Internet access at home	binary: yes or no
nursery	attended nursery school	binary: yes or no
higher	wants to take higher education	binary: yes or no
romantic	with a romantic relationship	binary: yes or no
freetime	free time after school	numeric: from 1—very low to 5—very high
goout	going out with friends	numeric: from 1—very low to 5—very high
Walc	weekend alcohol consumption	numeric: from 1—very low to 5—very high
Dalc	workday alcohol consumption	numeric: from 1—very low to 5—very high
health	current health status	numeric: from 1—very bad to 5—very good
absences	number of school absences	numeric: from 0 to 93
G1	first period grade	numeric: from 0 to 20
G2	second period grade	numeric: from 0 to 20
G3	final grade	numeric: from 0 to 20

**Table 2:** Performance of deep SVM without using improved CGAN.

Tutoring Method	Sensitivity	Specificity	AUC
School tutoring	91.8%	91.8%	90.9%
Home tutoring	92.2%	92.7%	91.1%
Combined tutoring	95.2%	94.7%	92.9%

**Table 3:** Performance of deep SVM when used with Improved CGAN.

Tutoring Method	Sensitivity	Specificity	AUC
School tutoring	95.1%	95.1%	93.2%
Home tutoring	91.9%	91.2%	91.4%
Combined tutoring	98.2%	97.1%	96.2%

**Table 4:** Performance of deep neural network models when used with Improved CGAN.

Tutoring Method	Sensitivity	Specificity	AUC
CNN			
School Tutoring	92.5%	94.3%	92.1%
Home Tutoring	89.9%	90.3%	90.7%
Combined Tutoring	97.3%	96.0%	96.0%
LSTM			
School Tutoring	90.4%	91.8%	90.1%
Home Tutoring	88.4%	89.4%	88.0%
Combined Tutoring	96.5%	94.3%	92.7%

**Table 5:** Results of machine learning models for school tutoring.

Model	Sensitivity	Specificity	AUC
RF	91.3	90.7	91.5
LR	92.2	93.4	92.8
ETC	91.3	94.1	91.8
GBM	88.4	89.3	87.2
SGD	89.6	89.5	90.5

**Table 6:** Results of machine learning models for home tutoring scenario.

Model	Sensitivity	Specificity	AUC
RF	88.3	88.2	88.1
LR	87.1	87.2	87.6
ETC	89.7	88.5	88.8
GBM	87.7	86.4	88.4
SGD	88.6	85.7	89.5

**Table 7:** Performance of models regarding combined tutoring.

Model	Sensitivity	Specificity	AUC
RF	94.1	95.4	94.5
LR	93.4	94.3	94.6
ETC	94.7	94.5	93.8
GBM	90.2	90.4	91.1
SGD	95.8	94.2	93.5

## Data Availability

The datasets generated during and/or analysed during the current study are publicly available. The link of the dataset is shared in the Section 4.1 of the manuscript. The dataset and code is available from the corresponding author on reasonable request.
